# Characterization of *Staphylococcus aureus* Isolates That Colonize Medical Students in a Hospital of the City of Cali, Colombia

**DOI:** 10.1155/2015/358489

**Published:** 2015-10-01

**Authors:** Luis Fernando Collazos Marín, Gina Estupiñan Arciniegas, Monica Chavez Vivas

**Affiliations:** ^1^Programa de Medicina, Facultad de Medicina, Universidad Santiago de Cali, Hospital San Juan de Dios de Cali, Carrera 4, No. 17-67, Cali, Colombia; ^2^Centro de Estudios e Investigacion en Salud (CEIS), Facultad de Salud, Universidad Santiago de Cali, Hospital San Juan de Dios de Cali, Carrera 4, No. 17-67, Cali, Colombia

## Abstract

*Introduction*. Nasal carriage of methicillin-resistant *Staphylococcus aureus* (MRSA) represents a risk for the spread of bacteria. This study characterized the *S. aureus* isolated from medical students, who were in their clinical rotation at a hospital in the city of Cali. *Materials and Methods*. 216 students participated in the study and 63 isolates of *S. aureus* were evaluated for susceptibility and PCR amplification of *agr* and *mec*A genes. The origin of MRSA isolates was established by analyzing *agr* polymorphisms. *Results*. A total of 29.2% of students were colonized by *S. aureus* and nasal carriage rate was 23.6% and 14.3% MRSA. Three *agr* groups (*agr* II, and *agr* III) were identified; the *agr* I group was the most common, with a 35% prevalence; this group is from community origin. *Conclusion*. The present study demonstrates that medical students carry *S. aureus* strains, with the threat of spreading them both to community and hospital environments.

## 1. Introduction

*Staphylococcus aureus* resistant to methicillin (MRSA) is currently among the bacteria of global concern [1, 2]. It is responsible for a broad range of community-associated MRSA infections, especially in young individuals soft tissue without risk factors [3–5] and at hospitals, specifically in intensive care units (ICU) [1, 6]. The most serious cases of MRSA resistance are those mediated by the* mec*A gene; these strains can simultaneously acquire pathogenicity genes that make them able to develop more aggressive infections [1].

Asymptomatic carriage of* S. aureus* in healthy individuals has been shown to have a high prevalence, especially in children, young adults, and healthcare workers [7–10]. Medical students represent an important portion of the healthcare staff, whom are in frequent contact with community in general or healthcare environment and may spread the bacteria to other community members or to susceptible patients, respectively [10–16].

Epidemiological studies conducted in Latin American hospitals report a prevalence of* S. aureus* colonization from 20 to 60% in health area students [10–13], with an important presence of MRSA strains in higher percentage than in Europe [14–16].

Epidemiological knowledge of the status of MRSA strains circulating in our environment helps to establish control measures to prevent the transmission of the pathogen through adequate use of biosafety barriers by the healthcare staff, including medical students. In this sense, two of the aspects that must be considered are whether the* S. aureus* carried by the healthcare staff is of intrahospital origin or from the community and how it is disseminated in hospital wards.

Molecular approaches, like analysis of the polymorphism in* agr* genes, have permitted determining the variability and origin of isolates generating epidemic outbreaks. According to this gene's variability pattern, to date, four variants have been defined, denominated as groups I to IV [17]. Isolates belonging to group III are related to infections associated with the community, while group II is detected predominantly in strains isolated at intrahospital level [18, 19].

The objective of this study was to establish the genetic diversity of isolates of* S. aureus* and detect the presence of the* mec*A gene in strains isolated from asymptomatic medical students who were in their clinical rotation phase in a hospital in the city of Cali.

## 2. Materials and Methods

### 2.1. Study Population

During the study, 481 medical students were in their preclinical and clinical rotation cycle; 216 students signed the informed consent and fulfilled with the inclusion and exclusion criteria, which included not having received antibiotic treatment within the last three months and not presenting respiratory or skin diseases. A total of 119 participants were women and 97 were men who were in hospital practices from October to December 2010 at the San Juan de Dios Hospital in the city of Cali. A cross-sectional descriptive study was carried out and it was evaluated and approved by the Ethics and Bioethics Committee of the Faculty of Health at the university.

### 2.2. Culture Conditions

Samples were taken with sterile cotton swabs through smears of the nasal mucosa and of the skin in each student. The samples were processed immediately in the microbiology laboratory. The isolates were managed by codes, thus, respecting student confidentiality.

To isolate species of the* Staphylococcus* genus, the samples were seeded in mannitol salt phenol red agar (Oxoid Ltd., Hampshire, United Kingdom) and incubated for 24 to 48 hours at 37°C. Identification of* S. aureus* was accomplished through fermentation of mannitol in selective agar (medium yellow coloration), the positive reaction of coagulase test, and microscope observation of Gram positive cocci in clusters from a direct extended Gram stain. The* S. aureus* was differentiated from coagulase-negative staphylococci (CoNS) by using the DNase test.

### 2.3. Antibiotic Susceptibility Tests

Antimicrobial susceptibility testing was conducted on paired samples using the agar disc diffusion method following the recommendations of the Clinical and Laboratory Standards Institute (CLSI) [20].

To conduct this test, a standardized amount of* S. aureus* was inoculated (standard 0.5 by Mc Farland) in Mueller-Hinton agar medium (Scharlau Chemie S.A.) and then the sensi-disks were placed: oxacillin (OXA, 1 *μ*g), cefoxitin (FOX, 30 *μ*g), cephalexin (LEX, 30 *μ*g), gentamicin (GEN, 10 *μ*g), ciprofloxacin (CIP, 5 *μ*g), erythromycin (ERY, 15 *μ*g), clindamycin (CLI, 2 *μ*g), trimethoprim/sulfamethoxazole (SXT 1.25/23.75 *μ*g), tetracycline (TET, 30 *μ*g), chloramphenicol (CHL, 30 *μ*g), vancomycin (VAN, 30 *μ*g), imipenem (IPM, 10 *μ*g), and penicillin (PEN, 10 U) (Oxoid). To verify the action of the sensi-disks, during susceptibility analysis the ATCC 25923 strain of* S. aureus* was used as control.

Determination of MRSA isolates was performed according to the results obtained in the evaluation of oxacillin and cefoxitin and by detecting the* mec*A gene [21, 22].

### 2.4. Molecular Analyses of Isolates

The DNA of the reference strains and of the bacterial isolates was extracted by using the modified protocol by Cheng and Jiang, based on bacterial lysis using 25% sucrose, 10 mg/mL of lysozyme, and 1 mg/mL of Proteinase K, at 56°C [23].

To establish resistance to methicillin, the* mec*A gene was amplified, following the protocol reported by Lee and colleagues using primers: MR1, 5′ (478)-GTG GAA TTG GCC AAT ACA GG-(497) 3′, and MR2, 5′ (1816)-TGA GTT CTG CAG TAC CGG AT-(1797) 3′ [22].

To determine the four* agr* groups, fragments of 440 bp, 572 bp, 406 bp, and 588 pb, respectively, were amplified independently using a set of primers, made up of the universal sense primer, with the* agr* gene sequence, 5′-ATG CAC ATG GTG CAC ATG C-3′, and one of the specific primers for each group in antisense,* agr* I 5′-GTC ACA AGT ACT ATA AGC TGC GAT-3′,* agr* II 5′-GTA TTA CTA ATT GAA AAG TGC CAT AGC-3,* agr* III 5′-CTG TTG AAA AAG TCA ACT AAA AGC TC-3′, and* agr* IV 5′-CGA TAA TGC CGT AAT ACC CG-3′ [18].

The PCR reactions were conducted in 50 *μ*L volume of a reaction mixture composed of MgCl_2_ 25 mM, 200 *μ*M of the four dNTPs, 0.5 U of Taq DNA polymerase (Invitrogen), 10 pmol of each primer, and 1 *μ*L of DNA solution in a thermocycler GeneAmp PCR system 2400. In this study* S. aureus* ATCC 25923 strain was used for the positive control.

### 2.5. Statistical Analysis

The unit of analysis was the bacterial isolate obtained from the nasal swab from which the microbiological and molecular characteristics were registered and were related with the sociodemographic characteristics of the carriers, like gender and academic semester. The microbiological variables were the presence of* S. aureus* and the degree of susceptibility or resistance to each antibiotic evaluated, categorized into different degrees, thus: resistance (1), intermediate susceptibility (2), and susceptibility (3), according to the standards for each antibiotic established for* S. aureus*. The molecular variables were presence of* mec*A genes and the variants of the* agr* gene. A database was constructed with the variables of interest by using the Excel program.

The presence of* S. aureus* was determined as percentage of time of permanence and gender. In the group colonized by* S. aureus*, an association analysis was performed among the different variables like resistance phenotype to different antibiotics, presence of* mec*A gene, and the* agr* genetic variant, bearing in mind the academic semester and the number of hours per day of practice: three hours for third-year students, five hours for fourth-year students, 8 hours for fifth-year students, and 12 hours for sixth-year students. Prevalence of the different* agr* gene variants was determined in MRSA and MSSA phenotypes.

The findings were statistically analyzed using descriptive statistics, Chi-square test (*χ* ^2^), and *P* value (*P* < 0.05, statistical significant). The risk factor analysis of MRSA colonization was performed using SPSS statistical package (version 20.0, SPSS, Inc., Chicago, IL, USA).

## 3. Results

A total of 29.2% (63) of the students who were in the different services at the hospital were colonized by* S. aureus*.  Table 1 shows the isolates according to the students' academic year; it can be noted that fourth-year students had the highest number of isolates (45%) (*P* < 0.05). The frequency of nasal carriage of* S. aureus* was significantly higher than on skin (20.4%* versus* 8.9%; OR = 2.870; *P* < 0.05) and fifth-year students presented the highest frequency of* S. aureus* in the nose than on the skin (18.2* versus* 1.8%; OR = 5.294; *P* = 0.094) (Table 1).

### 3.1. Antibiotic Susceptibility Analysis

Isolates with resistance to *β*-lactam antibiotics were distributed in a high number of students from all the academic grades, with a variation between 90 and 22% (Table 2). Isolates resistant to antibiotics inhibiting protein synthesis were registered between 18% and 90% and to antibiotics inhibiting nucleic acid synthesis registered between 11% and 44%.

### 3.2. Molecular Analysis of Isolates

Amplification of the* mec*A gene was detected in the five isolates presenting simultaneous resistance to oxacillin-cefoxitin and in four isolates resistant only to cefoxitin (14.3%). The multidrug resistance phenotype was the distinctive characteristic in these MRSA isolates with simultaneous resistance to *β*-lactam, macrolide, aminoglycoside, and quinolone antibiotics (Table 3).

A total of 54 isolates were positive to* agr* gene amplification with a difference in prevalence of specific* agr* groups between MRSA and methicillin sensitive* S. aureus* (MSSA) isolates. Thirty-five bacterial isolates (55.6%) were classified into* agr* group I, 31 (49.2%) strains were MSSA, and 4 (6.3%) strains were MRSA; the differences were statistically significant (*P* < 0.05).

Five MRSA isolates (9.3%) were identified in* agr* group II; the* mec*A gene was detected in all isolates and* agr* group III was found only in 14 MSSA isolates (22.2%) (Table 4).

The majority of* S. aureus* strains isolated from students belonged to* agr* group I followed by* agr* group III (41 strains), and finally* agr* group II (9 strains) was distributed among the isolates of fourth-, fifth-, and sixth-year students. The* agr* groups I to III occurred in the major percentages (25.9% and 18.5%, resp.) in fourth-year students (data not shown).

## 4. Discussion

The present study evidenced the presence of* S. aureus* with 29.2% of prevalence in medical students in clinical rotation, and nasal carriage was a risk factor. The tendency is similar in Latin American countries with percentages between 50 and 20% [10–13]. In Europe, a prevalence of nasal carriers of* S. aureus* is reported at 25% [14–16]. In Asia, prevalence of colonization by* S. aureus* in Chinese, Malaysian, and Indian medical students is registered in 31% of the cases [24].

Although some studies report increased frequency of nasal carriers as medical students have greater exposure to the hospital environment during clinical rotations [25, 26], our results did not show significant differences among the nasal carriers and the time of permanence in the hospital environment. However, a significant number of isolates resistant to imipenem were determined in third-year students and a higher number of isolates with resistance to aminoglycosides, erythromycins, and quinolones in students with greater time of permanence in the hospital (Table 2).

Our study determined a high frequency of* S. aureus* isolates resistant to penicillin, cephalexin, imipenem, erythromycin, chloramphenicol, clindamycin, tetracycline, gentamicin, trimethoprim-sulfamethoxazole, and ciprofloxacin, giving way to the presence of multidrug resistant isolates. These isolates can evolve in response to selective pressure exerted on microflora residing in patients, which are then passed on to students, or can be directly generated in the microflora of students.

Similar results were obtained in studies conducted by Lee and colleagues [22]; all isolates with resistance to cefoxitin presented the* mec*A gene. In their study, Datta and colleagues demonstrated that cefoxitin induces the expression of the PBP2a protein encoded in the* mec*A gene; the cefoxitin disk diffusion test had the best diagnostic performance among the phenotypic methods for detection of MRSA [21].

One of the factors that permit the presence of MRSA strains in asymptomatic carriers is due to antibiotic intake that strengthens overgrowth of organisms on the skin and on mucosa surface [2, 6]. It has been demonstrated that use of penicillin is associated with acquisition of MRSA; likewise, use of fluoroquinolones, macrolides, and cephalosporins increases the risk of having these strains [27].

Although in Colombia it is reported that the majority of MRSA strains are not of nosocomial origin [2], an increasing number of reports indicate an epidemiological change with the prevalence of strains of community origin [5, 28].

This study determined that strains of hospital origin (AH-MRSA) presented resistance to *β*-lactam antibiotics and to multiple antibiotics (Table 3) and, according to the* agr* gene analysis these isolates were located in* agr* group II, a group compatible with hospital origin [17, 18].

The presence of AH-MRSA strains in asymptomatic carriers is a potential risk of disseminating in the community; additionally, these strains are resistant to multidrug with few therapeutic alternatives.

However, we also found isolates with characteristics of community origin (AC-MRSA) in four MRSA isolates grouped in* agr* I. Nasal carriage of MRSA becomes an important reservoir and source of pathogen propagation among the personnel, the community, and the patient.

As observed in Table 3, MSSA isolates also presented resistance to multidrug characteristics. Studies conducted in Spain reveal that MSSA isolates susceptibility also shows resistance to erythromycin (13.5%), clindamycin (11.5%), and ciprofloxacin (1.9%) [16].

In addition, 49.2% and 22.2% of MSSA isolates were grouped in* agr* I and* agr* III, respectively, associating them with a molecular community origin.

Several studies have found that the MRSA and MSSA circulating in Colombia have genetic similarities to clone USA300 [29, 30]. This clone usually (ST8-MRSA-IVa) harbors the* sek*,* seq*,* bsaB*,* lukF-PV*, and* lukS-PV* genes, which encode the staphylococcal enterotoxins K and Q, bacteriocin, and Panton-Valentine leukocidin (PVL), respectively. Additionally, the clone typically contains a type I* agr* operon. The spread of MRSA could also be caused by the acquisition of* SCCmec* among asymptomatic carriers of* S. aureus* in the community [30]. Thus, the asymptomatic carriage of MSSA in medical students may contribute to the spread of MRSA between the community and hospitals.

Although some of the risk factors for colonization by* S. aureus* are age, gender, hospitalizations, use of antibiotics, and some diseases and habitat [3, 12], among the population analyzed, these variables were not evaluated, which became a limitation of the study. The results of this study, however, evidence the important presence of asymptomatic carriers* S. aureus* in medical students from the time they begin their clinical practice. Additionally, increased MRSA colonization was found in students as they spent more time in the hospital, as it is seen in fifth-year students showing the highest number of carriers.

## Figures and Tables

**Table 1:** Frequency of isolation of *S. aureus *from medical students.

Academic degree	Students		Students colonized						
	Total		* S. aureus*			OR	CI 95%		*P*
	*N*	%	*n* (%)	Nose *n* (%)	Skin *n* (%)		Min	Max	
3	31	14.4	9 (14.3)	7 (15.9)	2 (10.5)	1.608	0.302	8.569	0.575
4	69	31.9	31 (49.2)	18 (40.9)	13 (68.4)	0.322	0.102	0.998	0.045
5	55	25.5	11 (17.5)	10 (27.3)	1 (5.3)	5.294	0.627	44.707	0.094
6	61	28.2	12 (19)	9 (20.5)	3 (15.8)	1.371	0.327	5.755	0.665
Total	216	100	63 (29.2)	44 (20.4)	19 (8.9)	2.870	1.658	4.969	0.000

OR: odds ratio; CI: confidence intervals; statistical significance was based on *P* ≤ 0.05.

**Table 2:** Antibiotic susceptibility patterns of *S. aureus* isolates.

Academic degree (*N*)	Students Total		RT Hours	OXA *n* (%)	PEN *n* (%)	FOX *n* (%)	LEX *n* (%)	IPM *n* (%)	VAN *n* (%)	CIP *n* (%)	SXT *n* (%)	TCY *n* (%)	GEN *n* (%)	CLI *n* (%)	ERY *n* (%)	CHL *n* (%)
	*n*	%														
3	31	14.4	3	0 (0)	9 (100)	0 (0)	8 (88.9)	5^*∗*^ (5.6)	6 (66.7)	1 (11.1)	4 (44.4)	5 (22.2)	2 (22.2)	6 (66.7)	8 (33.3)	7 (55.6)
4	69	31.9	5	2 (6.5)	27 (87.1)	4 (12.9)	29 (93.5)	5 (16.1)	11 (35.5)	12 (38.7)	10 (23.3)	15 (23.3)	9 (30)	13^*∗*^ (23.3)	22 (50)	22 (46.7)
5	55	25.5	8	2 (18.2)	11 (100)	3 (27.3)	10 (90.9)	2 (18.2)	10^*∗*^ (90.9)	2 (18.2)	6 (54.5)	7 (63.3)	2 (18.2)	8 (72.7)	10 (90.9)	8 (72.7)
6	61	28.2	12	1 (8.3)	11 (91.7)	2 (16.7)	10 (83.3)	2 (16.7)	3 (25)	4 (33.3)	6 (50)	7 (58.3)	5 (45.5)	9 (75)	10 (83.3)	5^*∗*^ (41.7)
Total	216	100		5 (7.9)	58 (92.1)	9 (14.3)	57 (90.5)	14 (22.2)	30 (47.6)	19 (30.2)	26 (41.3)	34 (54)	18 (28.6)	36 (36.5)	50 (57.1)	42 (66.7)

RT: residence time of medical students in hospital. ^*∗*^ *P* ≤ 0.05.

*Note*. OXA, oxacillin; PEN, penicillin; FOX, cefoxitin; LEX, cephalexin; IPM, imipenem; VAN, vancomycin; CIP, ciprofloxacin; SXT, trimethoprim-sulfamethoxazole; TET, tetracycline; GEN, gentamicin; CLI, clindamycin; ERY, erythromycin; CHL, chloramphenicol.

**Table 3:** Frequency of MRSA and MSSA strains according of antibiotic susceptibility patterns.

Isolate	Antibiotic										
	PEN	FOX	IPM	VAN	CLI	ERI	CHL	GEN	TET	SXT	CIP
MRSA% (*n* = 9)	100 (9)	100 (9)	77.8 (7)	77.8 (7)	88.9 (8)	100 (9)	77.8 (7)	77.8 (7)	100 (9)	66.6 (6)	66.6 (6)

MSSA% (*n* = 54)	90.7 (49)	88.9 (48)	13 (7)	0 (0)	51.9 (28)	76 (41)	64.8 (35)	20.4 (11)	46.3 (25)	37 (20)	24.1 (13)

MRSA: methicillin-resistant *S. aureus*; MSSA: methicillin-sensitive *S. aureus*.

**Table 4:** Genetic polymorphism of the *agr* locus among MRSA and MSSA strains carried.

	*agr *I *n* (%)	*agr *II *n* (%)	*agr *III *n* (%)	*agr* (−) *n* (%)	Total *n*
MRSA	4 (6.3)	5 (7.9)	0	0	9
MSSA	31 (49.2)	0	14 (22.2)	9 (14.3)	54
Total	35 (55.6)	5 (7.9)	14 (22.2)	9 (14.3)	63
